# Vertical Control in Molar Distalization by Clear Aligners: A Systematic Review and Meta-Analysis

**DOI:** 10.3390/jcm13102845

**Published:** 2024-05-11

**Authors:** Tiffany H. Park, Christie Shen, Chun-Hsi Chung, Chenshuang Li

**Affiliations:** 1 School of Dental Medicine, University of Pennsylvania, Philadelphia, PA 19104, USA; thaparkk@upenn.edu; 2 Department of Orthodontics, School of Dental Medicine, University of Pennsylvania, Philadelphia, PA 19104, USA; chrishen@upenn.edu (C.S.); chunc@upenn.edu (C.-H.C.)

**Keywords:** aligners, class II, class III, intrusion, orthodontics, sequential distalization

## Abstract

**Background:** Molar distalization is used to correct molar relationships or to create space for mild anterior crowding. However, whether clear aligners can provide proper vertical control with the sequential distalization strategy has been highly debated. Thus, the current study aimed to systematically review the amount of dentoskeletal changes in the vertical dimension that results from sequential molar distalization in clear aligner therapy without temporary anchorage devices (TADs). **Methods:** Registered with PROSPERO (CRD42023447211), relevant original studies were screened from seven databases and supplemented by a manual search by two investigators independently. Articles were screened against inclusion and exclusion criteria, and a risk of bias assessment was conducted for each included article. Relevant data were extracted from the included articles and meta-analysis was performed using RStudio. **Results:** Eleven articles (nine for maxillary distalization and two for mandibular distalization) were selected for the final review. All studies have a high or medium risk of bias. For maxillary molar distalization, the meta-analysis revealed 0.26 mm [0.23 mm, 0.29 mm] of maxillary first molar intrusion based on post-distalization dental model analysis, as well as 0.50 mm [−0.78 mm, 1.78 mm] of maxillary first molar intrusion and 0.60 mm [−0.42 mm, 1.62 mm] of maxillary second molar intrusion based on post-treatment lateral cephalometric analysis. Skeletally, there was a −0.33° [−0.67°, 0.02°] change in the SN-GoGn angle, −0.23° [−0.30°, 0.75°] change in the SN-MP angle, and 0.09° [−0.83°, 1.01°] change in the PP-GoGn angle based on post-treatment lateral cephalometric analysis. There was insufficient data for meta-analysis for mandibular molar distalization. **Conclusions:** No significant changes in vertical dimension were observed, both dentally and skeletally, after maxillary molar distalization with a sequential distalization strategy. However, further studies on this topic are needed due to the high risk of bias in the currently available studies.

## 1. Introduction

Maxillary and mandibular molar distalization are commonly used strategies for correcting molar relationships, creating space for mild arch crowding or correcting bimaxillary protrusion [1]. For the maxillary arch, molar distalization has historically been achieved by the use of appliances such as the pendulum or headgear, while for mandibular molar distalization, class III elastics and multiloop edgewise archwire (MEAW) are more commonly used [2]. However, without the usage of temporary anchorage devices (TADs), undesirable dentoalveolar consequences may result from distalizing molars, including extrusion of posterior teeth [3], dental tipping [4], and loss of anterior anchorage [5,6]. In addition, even without dental extrusion, clockwise mandibular rotation and an increase in skeletal vertical dimension could occur due to the “wedge effect” that results when molars are distalized to the posterior alveolar region [7,8]. Thus, molar distalization should be used with caution, especially for hyperdivergent patients [9,10,11,12].

One of the more recent strategies for correcting molar relationships comes with the advent of clear aligner therapy. Due to its appearance and convenience, clear aligner therapy has grown in popularity amongst patients, especially adults [13]. Through sequential distalization strategy, clear aligners have been proven to achieve maxillary first molar distalization with a mean efficacy of 87% [14]. Regarding the efficacy of mandibular molar distalization, a systematic review suggests that 2–3 mm is possible for mandibular molar distalization with clear aligners in combination with TADs [15]. While the existing literature suggests promising implications of utilizing clear aligners to distalize molars, whether clear aligners can provide proper vertical control during molar distalization is still highly debated. Some clinicians state that by covering the occlusal surface of the maxillary and mandibular arches, the clear aligners function as posterior bite turbos, providing efficient molar intrusion [16,17,18]. However, this theory is not fully supported by others [19]. Especially during sequential distalization, the use of inter-arch elastics could extrude molars of the opposing arch [20,21] and increase the mandibular plane angle. Thus, the purpose of this study was to conduct a systematic review evaluating the size of dentoskeletal changes in the vertical dimension that result from sequential molar distalization in clear aligner therapy without TADs, and to provide clinical insight into the effectiveness and limitations when prescribing such treatments.

## 2. Materials and Methods

Registered with PROSPERO (registration number: CRD42023447211) on 1 August 2023, this study is compliant with the 2020 Preferred Reporting Items for Systematic Reviews and Meta-Analyses (PRISMA) guideline [22]. The following electronic databases were accessed for original articles: MEDLINE (PubMed), EBSCOhost, Web of Science, Elsevier (SCOPUS), Cochrane, LILACS (Latin American and Caribbean Health Sciences Literature), and Google Scholar. The literature search was finished on 19 January 2024.

### 2.1. Study Selection Criteria

**Figure 1:**
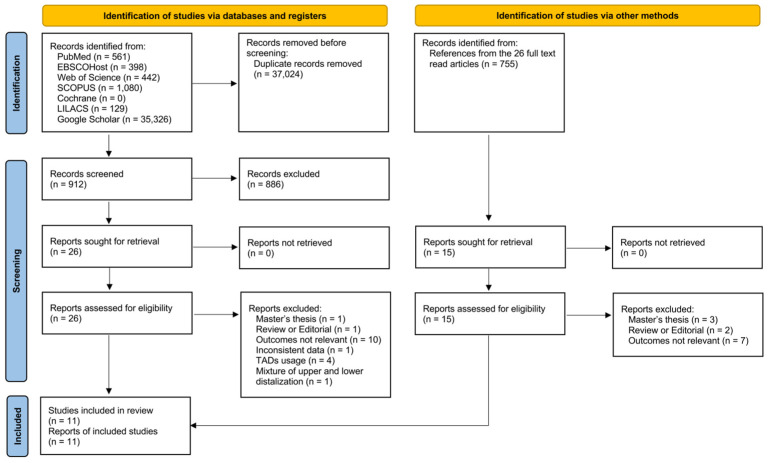
The PRISMA flow diagram demonstrating the study identification and screening.

Following the population, interventions, comparison, and outcome (PICO) outline, a systematic literature search was conducted regarding the effects of molar distalization with clear aligners on vertical dimension both dentally and skeletally (Table 1). The inclusion criteria were (1) longitudinal studies (both prospective and retrospective) comparing pre- and post-distalization/treatment records, (2) participants with permanent dentition, and (3) molar distalization achieved by sequential distalization strategy without TADs. The exclusion criteria were (1) participants with congenital abnormalities or systemic pathologies, (2) case reports, (3) conference abstracts, (4) opinions, editorials, or letters to the editors, and guidelines, (5) systematic reviews, (6) utilizing TADs or other auxiliaries during molar distalization, (7) no data reported about the dental or skeletal changes in the vertical dimension, and (8) inconsistent data within the manuscript. No language or date restrictions were applied. Figure 1 depicts the PRISMA flow diagram to obtain the final included articles.

### 2.2. Search Strategy

Our search strategy in all the used databases is as follows: (“aligners” AND “molar distalization”), (“aligner” AND “molar distalization”), (“clear aligners” AND “molar distalization”), (“clear aligner” AND “molar distalization”), (“sequential distalization”), (“class II” AND “aligners”), (“class II” AND “aligner”), (“class II” AND “clear aligner”), (“class II” AND “clear aligners”), (“class III” AND “aligners”), (“class III” AND “aligner”), (“class III” AND “clear aligner”), (“class III” AND “clear aligners”), and (“invisible removable thermoplastic appliance”). This search was supplemented by a manual search of the references listed in the articles that were included for full article reading. The full texts of the obtained articles were reviewed in detail and screened against the inclusion and exclusion criteria. Two authors (T.H.P. and C.S.) conducted the literature search and screening independently to ensure the reliability and completeness of the literature search results. When inconsistencies were encountered between the two authors, a third author was brought in for discussion (C.L.).

### 2.3. Data Extraction and Analysis

For all the articles finally included for further data analysis, relevant information was extracted from each article, including study type, sample size, gender, age, clear aligner brand, type of records, timing of treatment records, parameters evaluating molar changes in the vertical dimensions based on the dental model superimposition or lateral cephalometric analysis, and parameters evaluating the mandibular plane angle changes based on the lateral cephalometric analysis.

### 2.4. Risk of Bias/Quality Assessment

After modeling the risk-of-bias protocol established in another study [23], which shares a similar design to this study, 17 biases were evaluated into 4 categories: study design, study measurements, statistical analysis, and other (Table 2), which were scored by 2 authors (T.H.P. and C.S.) individually. A third author (C.L.) was consulted in the instances of disagreement. Individual article scores were determined by scoring the number of criteria met divided by the total number of criteria. Low, medium, or high risk of bias was determined based on randomization and reliability testing. A low risk of bias was determined if both reliability and randomization were met. A high risk of bias was determined if inter-rater reliability was not assessed and if randomization was not conducted. All other studies were determined as having a medium risk of bias (Table 2).

### 2.5. Statistical Analysis

The outcomes of the study were twofold: (1) the amount of dental vertical change following molar distalization by clear aligners; and (2) the amount of skeletal vertical change in the aspect of mandibular plane angle following molar distalization by clear aligners. Meta-analysis with the data from the included articles was conducted using RStudio (version 2023.09.1 + 494, Posit Software, PBC) [32,33]. For articles that only reported mean difference and upper and lower 95% confidence intervals (95% CI), the standard deviation was calculated using the definition of standard deviation [$SD=\sqrt{N}\times (upperlimit-lowerlimit)/3.92)$], regardless of the normal distribution of the sample population [34]. Meta-analysis was performed using a random-effects model and heterogeneity was assessed for variance between studies with the Tau2 method (τ^2^). Data are presented with mean and 95% CI. Sensitivity analysis and selective reporting within studies were not assessed due to the limited number of studies included per analyzed variable.

## 3. Results

### 3.1. Literature Searching and Study Selections

An initial search through seven electronic databases identified 37,936 potential articles (561 from PubMed, 398 from EBSCOHost, 442 from Web of Science, 1080 from SCOPUS, 0 from Cochrane, 129 from LILACS, and 35,326 from Google Scholar) (Figure 1). After duplicate records were removed, 912 articles remained for abstract screening. From the abstract reading, 886 articles were excluded, and 26 reports were retrieved for full-text reading. A total of 755 records were also manually retrieved from the references of these 26 articles, and 15 articles were retrieved for full-text reading in addition to the previous 26 reports.

Among the 41 reports, 30 were excluded because the articles were master theses [35,36,37,38], were reviews or editorials [39,40,41], outcomes were not relevant [14,42,43,44,45,46,47,48,49,50,51,52,53,54,55,56,57], had inconsistent data (and could not get the responses from the corresponding author) [58], utilized TADs [59,60,61,62], or had a mixture of upper and lower distalization [63]. Therefore, after adhering to the guidelines presented by the PRISMA, eleven articles were included for final analysis [6,12,17,24,25,26,27,28,29,30,31].

### 3.2. Risk of Bias

The strength of evidence was assessed by performing a methodological risk of bias assessment on the eleven included studies (Table 2). Only one study [24] reported both random sampling and random allocation of treatment, whereas another study [29] reported only random sampling in their study. The rest of the included studies did not report randomization. Four studies reported blinding completed by the examiner [6,17,24,28] but only one of those studies [24] completed blinding by the statistician. The other studies did not include blinding measurements. Intra-rater reliability was reported in five of the eleven studies [17,24,28,29,30]. One article [26] was unclear with its reporting of intra-rater reliability. Only one article [17] reported inter-rater reliability. Overall, no study scored low for risk of bias. Three studies [17,24,29] had a medium risk of bias score whereas the other eight [6,12,25,26,27,28,30,31] had a high overall risk of bias.

### 3.3. Demographic Data

The main characteristics of the included studies are summarized in Table 3. Three of the studies were prospective [24,29,31], five were retrospective [6,12,17,28,30], and three were unclear about their study type [25,26,27]. Most of the studies utilized Invisalign as their choice of clear aligner but one article by Zhang et al. [26] used Angel Aligner, and the article by Cui et al. [12] was unclear about the clear aligner brand used. There were nine articles that used the sequential distalization strategy to distalize the maxillary molars with 138 subjects in total [12,17,24,25,26,27,28,29,30], and two articles that used sequential distalization to distalize the mandibular molars with 36 subjects in total [6,31]. The overall sample population comprised late adolescents and adults.

Regarding the type and timing of treatment records, high heterogeneity was noticed (Table 3). For instance, there were three articles that performed evaluations based on post-distalization (only after molars being distalized) records: two with digital dental models [25,26] and one with cone-beam computed tomography (CBCT) images [31]; eight articles performed evaluations based on post-treatment records: one with both digital models and lateral cephalometric X-rays [27], one with both digital models and CBCT [30], five with lateral cephalometric X-rays only [6,17,24,28,29], and one with CBCT only [12].

Thus, the following data collection and analysis are sub-grouped based on the arch that distalization was being performed on and the timing and type of records provided in each included article.

### 3.4. Dental Vertical Changes from Maxillary Molar Distalization

**Figure 2:**
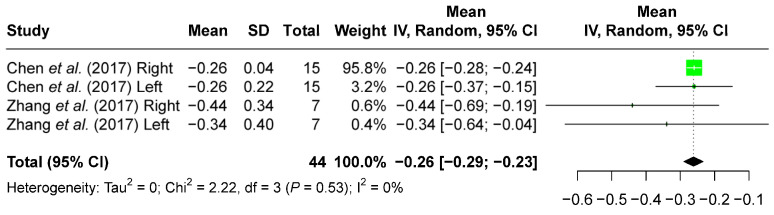
Forest plot of the amount of maxillary first molar vertical changes evaluated from the superimposition of pre-treatment and post-distalization dental models after maxillary molar distalization [25,26].

The amount of dental vertical control evaluated from dental models following maxillary molar distalization is summarized (Table 4), which shows the overall trend of slight maxillary first and second molar intrusion at both post-distalization and post-treatment time points. Limited by the number of available articles on this aspect, a meta-analysis could only be performed for the maxillary first molar changes evaluated on the post-distalization dental models. A random-effects model of meta-analysis revealed a minimal amount of maxillary first molar intrusion (−0.26 mm [−0.29 mm, −0.23 mm]) by clear aligners after sequential distalization (Figure 2).

Additionally, the amount of dental vertical control after maxillary molar distalization evaluated from lateral cephalometric radiographs is summarized in Table 5. Only data for the post-treatment timepoint were available. The included articles recorded the amount of intrusion or extrusion based on cusp or root reference points from the maxillary first and second molar in relation to the occlusal plane or palatal plane. Since the occlusal plane changes based on the position of the molars, further analysis focused on the relationship between the molars and the palatal plane.

**Figure 3:**
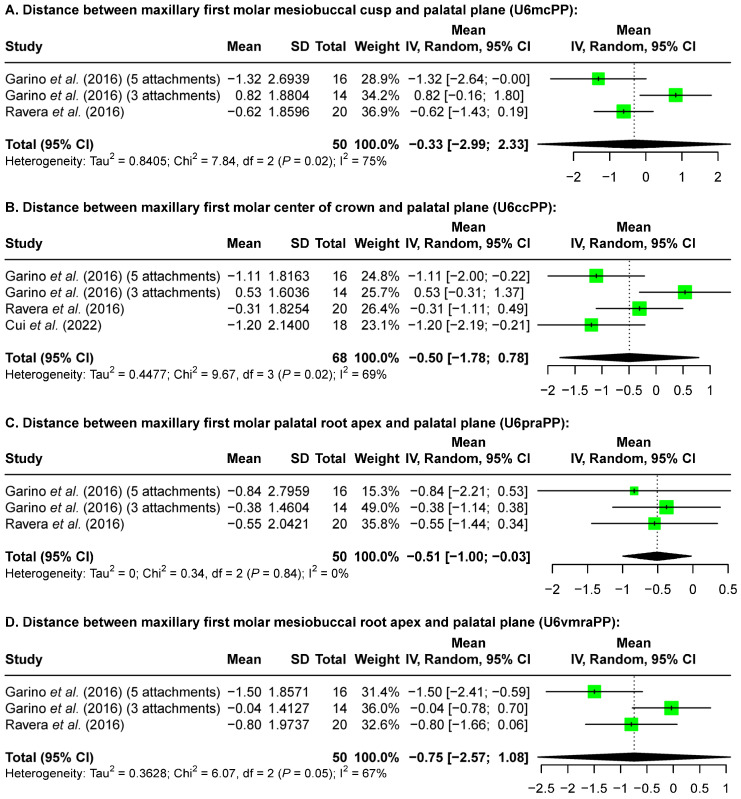
Forest plots of the size of maxillary first molar vertical changes after maxillary molar distalization according to pre- and post-treatment lateral cephalometric analysis. A positive value indicates molar extrusion while a negative value indicates molar intrusion [12,17,24].

**Figure 4:**
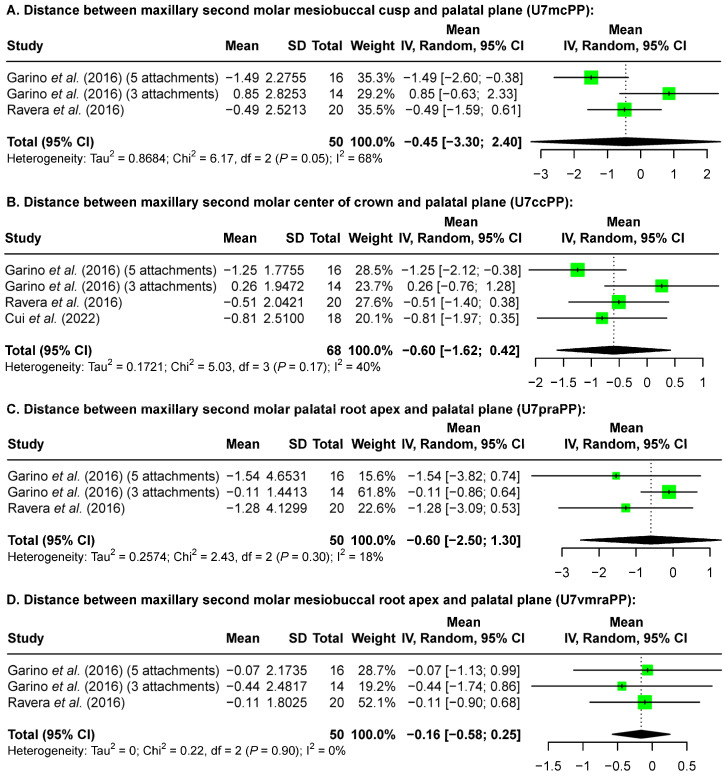
Forest plots of the size of maxillary second molar vertical changes after maxillary molar distalization according to pre- and post-treatment lateral cephalometric analysis. A positive value indicates molar extrusion while a negative value indicates molar intrusion [12,17,24].

While a definite trend cannot be determined from the range of reported changes (Table 5), a meta-analysis using a random-effects model overall revealed no vertical position change of the maxillary first (Figure 3) and second molars (Figure 4). Specifically, at the crown level, the maxillary first molar mesiobuccal cusp in relation to the palatal plane demonstrated −0.33 mm [−2.99 mm, 2.33 mm] vertical change (Figure 3A) and the center of the crown to the palatal plane showed −0.50 mm [−1.78 mm, 0.78 mm] (Figure 3B); at the root level, the distance between the maxillary first molar palatal root apex and the palatal plane showed intrusion of −0.51 mm [−1.00 mm, −0.03 mm] (Figure 3C), while the mesiobuccal root apex showed −0.75 mm [−2.57 mm, 1.08 mm] (Figure 3D). Similarly, the maxillary second molar mesiobuccal cusp in relation to the palatal plane demonstrated a change of −0.45 mm [−3.30 mm, 2.40 mm] (Figure 4A), and the center of the crown of the maxillary second molar to the palatal plane showed a change of −0.60 mm [−1.62 mm, 0.42 mm] (Figure 4B). The distance between the maxillary second molar palatal root apex and the palatal plane showed a change of −0.60 mm [−2.50 mm, 1.30 mm] (Figure 4C), while the mesiobuccal root apex showed −0.16 mm [−0.58 mm, 0.25 mm] (Figure 4D).

### 3.5. Skeletal Vertical Changes from Maxillary Molar Distalization

The amount of skeletal vertical control evaluated from lateral cephalometric radiographs following maxillary molar distalization is summarized (Table 6). Only data from the post-treatment time point were available. The data related to the mandibular plane were collected, but large variations among studies were noted.

**Figure 5:**
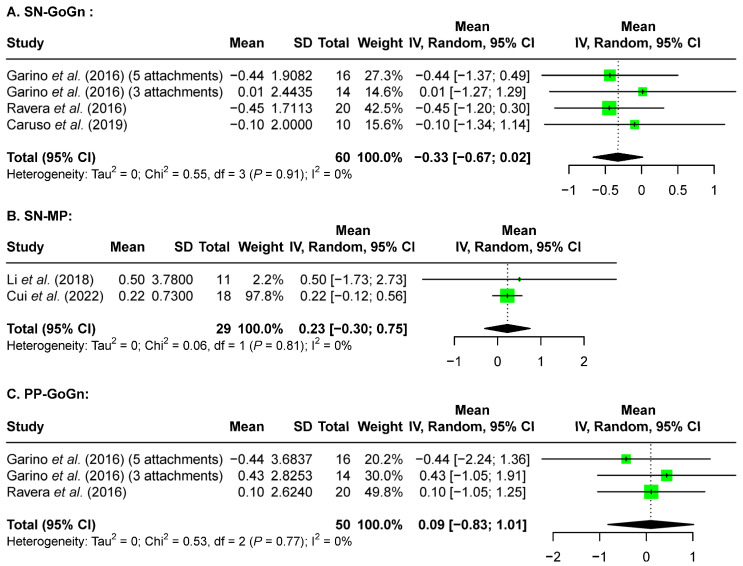
Forest plots of the size of skeletal vertical changes after maxillary molar distalization according to pre- and post-treatment lateral cephalometric analysis. A positive value indicates an increase in the mandibular plane angle after treatment, and a negative value indicates a decrease in the mandibular plane angle after treatment [12,17,24,27,28].

A random-effects model was used for meta-analysis and showed a change of −0.33° [−0.67°, 0.02°] for the SN-GoGn (sella-nasion ^ Gonion-gnathion) angle (Figure 5A), 0.23° [−0.30°, 0.75°] for the SN-MP (sella-nasion ^ mandibular plane) angle (Figure 5B), and 0.09° [−0.83°, 1.01°] for the PP-GoGn (palatal plane ^ gonion-gnathion) angle (Figure 5C). Thus, no significant changes were observed in the skeletal parameters.

### 3.6. Dental Vertical Changes from Mandibular Molar Distalization

While the number of articles that report vertical changes from mandibular molar distalization using sequential distalization is few, one article presented the size of mandibular first and second molar vertical changes using 3D valuations on CBCT at the time point of post-distalization [31]. All the landmarks utilized in this study reported 0.29–1.06 mm intrusion of mandibular first and second molars based on the mean values of each parameter, but large standard deviations were noticed (Table 7).

### 3.7. Skeletal Vertical Changes from Mandibular Molar Distalization

Two articles reported the size of skeletal vertical changes evaluated from lateral cephalometric analysis following mandibular molar distalization with sequential distalization (Table 8). For both post-distalization and post-treatment records, the included studies demonstrate an overall trend of an increase in the mandibular plane angle. Only Wu et al. [31] reported that the SN-MP angle from post-distalization records showed a decrease of −0.99°. Large standard deviations or large ranges of the 95% confidence interval were also noticed from the reported parameters.

## 4. Discussion

### 4.1. Summary of Evidence

Clear aligner therapy in orthodontics has experienced a surge in recent decades. Besides the advantages of aesthetics, comfort, and oral hygiene maintenance for patients, some clinicians claim that one of the clinical benefits of clear aligners is vertical control due to the “bite block” effects [64,65,66]. In fact, vertical control has been a challenging problem in orthodontics, especially for patients with a hyperdivergent skeletal pattern, because fixed appliances tend to extrude the teeth and cause clockwise rotation of the mandible [67]. In addition, when molars are distalized into the wedge of the occlusion, clockwise rotation of the mandibular plane is further introduced despite maintaining the molars in the same vertical position [7]. Thus, better vertical control during molar distalization implies the opportunity for predictable success with clear aligners and less room for detrimental side effects. However, there is no solid evidence to confirm such claims. The articles in this study, therefore, shed more light on the ongoing conversation about vertical control following molar distalization with the sequential distalization strategy of clear aligner therapy.

During the literature search and analysis, high heterogeneity was noticed regarding the time points and types of record utilized in each study. Such heterogeneity significantly increased the complexity of data analysis and reduced the amount of data that could be utilized for each meta-analysis. To perform a meta-analysis, data stratification based on the arch, timepoint, and type of record need to match. In addition, none of the included studies performed a comparison between clear aligner therapy and fixed appliances. Consequently, no direct evidence could be provided regarding which type of appliance provides better vertical control during molar distalization.

Through meta-analysis, our study revealed 0.26 mm [0.23 mm, 0.29 mm] maxillary first molar intrusion based on the pre-treatment and post-distalization dental model superimposition after maxillary molar distalization (Figure 2). While the post-treatment timepoint revealed no significant vertical change from the landmarks on the crown of the maxillary first molars, a slight intrusion of the palatal root apex of the maxillary first molar (−0.51 mm [−1.00 mm, −0.03 mm]) was observed (Figure 3). The differences between these two time points may be the result of several factors. First, the molar position may continue to change from post-distalization to post-treatment time points. Second, the use of different types of records also indicates the use of different reference landmarks. Third, tracing errors may have occurred from distortion, magnification, and overlapping structures on the lateral cephalometric X-rays. Such errors are likely, as different results were found at the crown and root levels when a meta-analysis was performed with data from the same studies (Figure 3A,C). These findings also suggest that caution needs to be taken when comparing the treatment effects of different appliances among studies with variant types of records.

Skeletally, no significant changes were observed with the SN-GoGn angle, SN-MP angle, and PP-GoGn angle (Figure 5), which indicate proper skeletal vertical control during the orthodontic treatment for maxillary molar distalization with clear aligner sequential distalization strategy.

Patients with a hyperdivergent skeletal pattern have weaker bite force and muscle efficiency than patients with a hypodivergent skeletal pattern [68]. As a result, the molar intrusion effects from the clear aligners may be less efficient in patients with a hyperdivergent pattern than in patients with a hypodivergent one. A previous study shows that the unplanned maxillary intrusion after clear aligner treatment was negatively associated with the mandibular plane angle [16]. However, molar intrusion is more favorable in hyperdivergent patients and molar extrusion is more favorable in hypodivergent patients during orthodontic treatment. Thus, it would be more critical for clear aligners to provide proper vertical control during sequential distalization in patients with a hyperdivergent pattern. For the studies included in the current review, none considered the influence of the skeletal vertical pattern on treatment effects. From the literature search, only one available study compared the efficiency of vertical control from clear aligners with sequential distalization in patients with different skeletal vertical patterns [63]. Interestingly, this study stated that there was a slight but not significant increase in the mandibular plane angle in low- and normo-angle patients and a decrease in the mandibular plane angle in high-angle patients after clear aligner treatment with sequential distalization [63]. However, this study, which comprised a mixture of samples with both maxillary and mandibular molar distalization as well as a large age range (10–53 years old), had an unclear sample distribution among three vertical pattern groups [63]. In addition, all three groups showed an increase in lower anterior facial height (ANS-Me) [63], making it unclear whether the changes in SN-MP angle were directly related to the skeletal vertical pattern of the patients. Thus, further studies in this aspect are required.

### 4.2. Limitations

To cautiously consider the results of this study as well as plan future studies, several limitations must be considered. First, out of the included studies, only one article stratified the respective results based on attachment design [24]. In fact, by comparing a group that had five attachments per quadrant to a group that had only three attachments per quadrant, Garino et al. stated that the five-attachment protocol provided better vertical control than the three-attachments protocol during maxillary molar distalization. However, not all the included studies provided information about attachment design. For the included studies that did report attachment design [17,25,27,28,29], different numbers of attachments ranging from 3 to 5 per quadrant were described. Even when the same number of attachments was used, different teeth were included. Therefore, directly combining studies from different groups with potentially different designs on attachments may overlook the true effects of clear aligners with different mechanical setups.

Second, it is unclear if specific amounts of active molar intrusion were programmed in the digital setups of each study. For instance, some studies may have built in a specific amount of molar intrusion into the digital setup to enhance the vertical control during molar distalization as indicated in the study from Peng et al. [63], whereas some studies had minimum vertical movement built into the clincheck but obtained more molar intrusion than predicted [30]. Thus, whether the 0.26 mm of molar instruction observed in the post-distalization model was from the complete or incomplete expression of molar intrusion built in to the digital setup, or from the bite block effects of the clear aligners [16,65], remains unclear.

Lastly, although the current research did not discuss the amount of distalization achieved in each study, a potential correlation between the amount of distalization and resulting vertical changes should be explored. Future studies can also incorporate comparisons between clear aligners and other molar distalization strategies regarding vertical control, ultimately aiming to enhance clinical judgment and decision-making for treatment.

## 5. Conclusions

This study demonstrates that no significant changes in vertical dimension were observed, both dentally and skeletally, after maxillary molar distalization with clear aligner sequential distalization. However, the high risk of bias in the currently available studies, the large variation in the measurement protocol among the studies, and the limited availability of evaluations of vertical changes after mandibular molar distalization imply the need for additional studies to confirm the efficiency of vertical control following molar distalization with clear aligners. In addition, future studies can categorize the vertical pattern of patients, as well as correlate the potential relationship between the amount of molar distalization and subsequent vertical control using clear aligners.

## Figures and Tables

**Table 1:** The PICO questions of this study.

Criteria	Description
Population	Patients undergoing orthodontic treatment with clear aligners requiring molar distalization
Intervention	Molar distalization with sequential distalization protocol of clear aligner therapy
Comparisons	The control is pre-treatment models and X-rays
Outcome	The amount of dental and skeletal vertical changes introduced by molar distalization with clear aligners

**Table 2:** Risk of bias assessment of the eleven included studies. +: Low risk of bias; ?: Medium risk of bias; -: High risk of bias. Highlight colors were used to help visualize the different sub categories within the key criterias that were used for determining the Risk of Bias (randomization and reliability testing).

		Maxillary									Mandibular	
		Garino et al. (2016) [24]	Ravera et al. (2016) [17]	Chen et al. (2017) [25]	Zhang et al. (2017) [26]	Li et al. (2018) [27]	Caruso et al. (2019) [28]	Cui et al. (2022) [12]	Balboni et al. (2023) [29]	Lin et al. (2023) [30]	Wu et al. (2021) [31]	Rota et al.(2022) [6]
1. Study Design (6)	A. Objective: objective clearly formulated	⊕	⊕	⊕	⊕	⊕	⊕	⊕	⊕	⊕	⊕	⊕
	B. Sample size: considered adequate and estimated before collection of data	⊕	⊕	?	⊖	?	?	?	⊕	⊖	?	⊕
	C. Baseline characteristics—similar baseline characteristics	⊕	⊕	?	?	⊕	?	⊕	⊕	⊕	⊕	⊕
	D. Co-interventions	⊕	⊕	⊕	⊕	⊕	⊕	⊕	⊕	⊕	⊕	⊕
	E. Randomization											
	Random Sampling	⊕	⊖	⊖	⊖	⊖	⊖	⊖	⊕	⊖	⊖	⊖
	Random Allocation of Treatment	⊕	⊖	⊖	⊖	⊖	⊖	⊖	⊖	⊖	⊖	⊖
2. Study Measurements (5)	F. Measurement method—appropriate to the objective	⊕	⊕	⊕	⊕	?	⊕	⊕	⊕	⊕	⊕	⊕
	G. Blind measurement—blinding											
	Blinding (examiner)	⊕	⊕	⊖	⊖	⊖	⊕	⊖	⊖	⊖	⊖	⊕
	Blinding (statistician)	⊕	⊖	⊖	⊖	⊖	⊖	⊖	⊖	⊖	⊖	⊖
	H. Reliability											
	Reliability described? (Intra-rater reliability)	⊕	⊕	⊖	?	⊖	⊕	⊖	⊕	⊕	⊖	⊖
	Adequate level of agreement? (Inter-rater reliability)	⊖	⊕	⊖	⊖	⊖	⊖	⊖	⊖	⊖	⊖	⊖
3. Statistical analysis (5)	I. Statistical analysis											
	Appropriate for data	⊕	⊕	⊕	⊖	⊖	⊕	⊕	⊕	⊕	⊖	⊕
	Combined subgroup analysis	⊕	⊕	⊕	⊖	⊖	⊕	⊕	⊕	⊕	⊖	⊕
	J. Cofounders (co-interventions)—confounders included in analysis	⊕	⊕	⊖	⊖	⊖	⊕	⊕	⊕	⊕	⊖	⊕
	K. Statistical significance level											
	*p* value stated?	⊕	⊕	⊕	⊖	⊕	⊕	⊕	⊕	⊕	⊕	⊕
	Confidence Intervals stated?	⊖	⊕	⊖	⊖	⊖	⊖	⊖	⊕	⊖	⊖	⊕
4. Other	L. Clinical significance	⊕	⊕	⊕	?	⊕	⊕	⊕	⊕	⊕	⊕	⊕
	Total Score	15	14	7	3	5	10	9	13	10	6	12
	Percentage of the Total	88.24	82.35	41.18	17.65	29.41	58.82	52.94	76.47	58.82	35.29	70.59
Risk of Bias		MED	MED	HIGH	HIGH	HIGH	HIGH	HIGH	MED	HIGH	HIGH	HIGH

**Table 3:** Characteristics of included studies. Max: maxillary; Mand: mandibular; F: female; M: male; CBCT: Cone-beam computed tomography. Y: yes; N: no.

Study	Maxillary or Mandibular Molar Distalization	Study Type	Age (Years)	Sample Size (F/M)	Clear Aligner Brand	Post-Distalization Records			Post-Treatment Records		
						Digital Model	Lateral Ceph	CBCT	Digital Model	Lateral Ceph	CBCT
Garino et al. (2016) [24]	Max	Prospective	30.5	30 (18F/12M)	Invisalign	-	-	-	N	Y	N
Ravera et al. (2016) [17]	Max	Retrospective	29.73 ± 6.89	20 (11F/9M)	Invisalign	-	-	-	N	Y	N
Chen et al. (2017) [25]	Max	Unclear	25.3 (14–43)	15	Invisalign	Y	N	N	-	-	-
Zhang et al. (2017) [26]	Max	Unclear	14.0 ± 3.1	7 (5F/2M)	Angel Aligner	Y	N	N	-	-	-
Li et al. (2018) [27]	Max	Unclear	25.3 (21–34)	11 (7F/4M)	Invisalign	-	-	-	Y	Y	N
Caruso et al. (2019) [28]	Max	Retrospective	22.7 ± 5.3	10 (8F/2M)	Invisalign	-	-	-	N	Y	N
Cui et al. (2022) [12]	Max	Retrospective	27.8 ± 5.38 (18–38)	18	Unclear	-	-	-	N	N	Y
Balboni et al. (2023) [29]	Max	Prospective	17.1 ± 3.2	20 (13F/7M)	Invisalign	-	-	-	N	Y	N
Lin et al. (2023) [30]	Max	Retrospective	26.64 ± 3.02 (23.1–31.5)	7	Invisalign	-	-	-	Y	N	Y
Wu et al. (2021) [31]	Mand	Prospective	>18	20 (12F/8M)	Invisalign	N	N	Y	-	-	-
Rota et al. (2022) [6]	Mand	Retrospective	25.6 ± 4.5	16 (8F/8M)	Invisalign	-	-	-	N	Y	N

**Table 4:** The size of dental vertical changes evaluated on the dental models after maxillary molar distalization. The data are presented as mean ± standard deviation. A positive value indicates molar extrusion, while a negative value indicates molar intrusion.

Time Points	Parameters		References	Changes (mm)
Post-Distalization	Maxillary First Molar (U6s)		Chen et al. (2017) [25] Right	−0.26 ± 0.04
			Chen et al. (2017) [25] Left	−0.26 ± 0.22
			Zhang et al. (2017) [26] Right	−0.44 ± 0.34
			Zhang et al. (2017) [26] Left	−0.34 ± 0.41
	Maxillary Second Molar (U7s)		Chen et al. (2017) [25] Right	−0.36 ± 0.34
			Chen et al. (2017) [25] Left	−0.37 ± 0.46
Post-Treatment	Maxillary First Molar (U6s)	MB cusp	Lin et al. (2023) [30]	−0.36 ± 0.66
		DB cusp	Lin et al. (2023) [30]	−0.36 ± 0.62
		MP cusp	Lin et al. (2023) [30]	0.01 ± 0.68

**Table 5:** The size of dental vertical changes evaluated on the lateral cephalometric radiograph after maxillary molar distalization. mcOP: distance between maxillary molar mesiobuccal cusp and occlusal plane; ccOP: distance between the maxillary molar center of crown and occlusal plane; praOP: distance between maxillary molar palatal root apex and occlusal plane; vmraOP: distance between maxillary molar mesiobuccal root apex and occlusal plane; mcPP: distance between maxillary molar mesiobuccal cusp and palatal plane, ccPP: distance between maxillary molar center of crown and palatal plane, praPP: distance between maxillary molar palatal root apex and palatal plane, vmraPP: distance between maxillary molar mesiobuccal root apex and palatal plane; UMVD: maxillary first molar vertical dimension. The data are either presented as a mean [95% confidence interval] or mean ± standard deviation. A positive value indicates molar extrusion while a negative value indicates molar intrusion.

Time Points	Parameters		References	Changes (mm)
Post-Treatment	Maxillary Firs Molar (U6s)	U6ccOP	Garino et al. (2016) [24] (5 attachments)	0.08 [−0.55, 0.72]
			Garino et al. (2016) [24] (3 attachments)	−0.37 [−1.01, 0.26]
			Ravera et al. (2016) [17]	0.05 [−0.46, 0.55]
		U6praOP	Garino et al. (2016) [24] (5 attachments)	0.32 [−0.72, 1.37]
			Garino et al. (2016) [24] (3 attachments)	−1.44 [−2.52, −0.37]
			Ravera et al. (2016) [17]	−0.24 [−0.90, 0.43]
		U6vmraOP	Garino et al. (2016) [24] (5 attachments)	−0.30 [−1.23, 0.64]
			Garino et al. (2016) [24] (3 attachments)	−1.12 [−2.07, −0.16]
			Ravera et al. (2016) [17]	−0.48 [−1.36, 0.41]
		U6mcPP	Garino et al. (2016) [24] (5 attachments)	−1.32 [−2.07, 0.57]
			Garino et al. (2016) [24] (3 attachments)	0.82 [−0.17, 1.80]
			Ravera et al. (2016) [17]	−0.62 [−1.44, 0.19]
			Caruso et al. (2019) [28]	−2.00 *
			Balboni et al. (2023) [29]	−0.9
		U6ccPP	Garino et al. (2016) [24] (5 attachments)	−1.11 [−2.00, −0.22]
			Garino et al. (2016) [24] (3 attachments)	0.53 [−0.31, 1.37]
			Ravera et al. (2016) [17]	−0.31 [−1.11, 0.49]
			Cui et al. (2022) [12] ^&^	−1.20 ± 2.14
		U6praPP	Garino et al. (2016) [24] (5 attachments)	−0.84 [−2.21, 0.53]
			Garino et al. (2016) [24] (3 attachments)	−0.38 [−1.14, 0.39]
			Ravera et al. (2016) [17]	−0.55 [−1.45, 0.34]
		U6vmraPP	Garino et al. (2016) [24] (5 attachments)	−1.50 [−2.41, −0.59]
			Garino et al. (2016) [24] (3 attachments)	−0.04 [−0.78, 0.70]
			Ravera et al. (2016) [17]	−0.80 [−1.67, 0.06]
		UMVD	Li et al. (2018) [27]	−0.95 ± 1.22
	Maxillary Second Molar (U7s)	U7mcOP	Garino et al. (2016) [24] (5 attachments)	0.06 [−0.58, 0.71]
			Garino et al. (2016) [24] (3 attachments)	0.14 [−0.35, 0.62]
			Ravera et al. (2016) [17]	0.29 [−0.23, 0.80]
		U7ccOP	Garino et al. (2016) [24] (5 attachments)	−0.01 [−0.93, 0.92]
			Garino et al. (2016) [24] (3 attachments)	−0.45 [−1.03, 0.13]
			Ravera et al. (2016) [17]	−0.01 [−0.74, 0.72]
		U7praOP	Garino et al. (2016) [24] (5 attachments)	0.33 [−0.52, 1.17]
			Garino et al. (2016) [24] (3 attachments)	−1.12 [−2.38, 0.14]
			Ravera et al. (2016) [17]	−0.13 [−1.09, 0.82]
		U7vmraOP	Garino et al. (2016) [24] (5 attachments)	−0.11 [−2.26, 2.04]
			Garino et al. (2016) [24] (3 attachments)	−1.16 [−2.48, 0.16]
			Ravera et al. (2016) [17]	−0.44 [−2.25, 1.27]
		U7mcPP	Garino et al. (2016) [24] (5 attachments)	−1.49 [−2.60, −0.37]
			Garino et al. (2016) [24] (3 attachments)	0.85 [−0.63, 2.33]
			Ravera et al. (2016) [17]	−0.49 [−1.59, 0.62]
			Caruso et al. (2019) [28]	−3.00 *
		U7ccPP	Garino et al. (2016) [24] (5 attachments)	−1.25 [−2.12, −0.38]
			Garino et al. (2016) [24] (3 attachments)	0.16 [−0.86, 1.18]
			Ravera et al. (2016) [17]	−0.51 [−1.40, 0.39]
			Cui et al. (2022) [12] ^&^	−0.81 ± 2.51
		U7praPP	Garino et al. (2016) [24] (5 attachments)	−1.54 [−3.82, 0.74]
			Garino et al. (2016) [24] (3 attachments)	−0.11 [−0.86, 0.65]
			Ravera et al. (2016) [17]	−1.28 [−3.09, 0.53]
		U7vmraPP	Garino et al. (2016) [24] (5 attachments)	−0.07 [−1.13, 1.00]
			Garino et al. (2016) [24] (3 attachments)	−0.44 [−1.04, 1.56]
			Ravera et al. (2016) [17]	−0.11 [−0.90, 0.68]

*: Data calculated based on post-treatment mean value—pretreatment mean value provided in the article. ^&^: three-dimensional cephalometric analysis on CBCT.

**Table 6:** The size of skeletal vertical changes evaluated on the lateral cephalometric radiographs after maxillary molar distalization. SN-GoGn: sella-nasion-gonion-gnathion, SN-MP: sella-nasion-mandibular plane angle, PP-GoGn: palatal plane-gonion-gnathion angle, FMA (FH-MP): Frankfurt horizontal-mandibular plane angle, Ar-Go-Me: articulare-gonion-menton angle (gonial angle). The data are presented as either mean [95% confidence interval] or mean ± standard deviation. A positive value indicates an increase in the mandibular plane angle after treatment, negative value indicates decrease in the mandibular plane angle after treatment.

Time Points	Parameters	References	Change (°)
Post-Treatment	SN-GoGn	Garino et al. (2016) [24] (5 attachments)	−0.44 [−1.37, 0.50]
		Garino et al. (2016) [24] (3 attachments)	0.01 [−1.28, 1.28]
		Ravera et al. (2016) [17]	−0.45 [−1.20, 0.30]
		Caruso et al. (2019) [28]	−0.1 ± 2.0
		Balboni et al. (2023) [29]	−0.3
		Lin et al. (2023) [30] ^+^	0.51 *
	SN-MP	Li et al. (2018) [27]	0.50 ± 3.78
		Cui et al. (2022) [12] ^&^	0.22 ± 0.73
	PP-GoGn	Garino et al. (2016) [24] (5 attachments)	−0.44 [−2.24, 1.37]
		Garino et al. (2016) [24] (3 attachments)	0.43 [−1.05, 1.91]
		Ravera et al. (2016) [17]	0.10 [−1.05, 1.25]
	FMA (FH-MP)	Li et al. (2018) [27]	1.56 ± 3.15
		Balboni et al. (2023) [29]	−1.3
	Ar-Go-Me	Balboni et al. (2023) [29]	−3.4

*: Data calculated based on post-treatment mean value—pretreatment mean value provided in paper. ^&^: three-dimensional cephalometric analysis on CBCT. ^+^: the authors of this article considered SN-GoGn and SN-MP as one parameter.

**Table 7:** The amount of dental vertical changes evaluated on radiographic images following mandibular molar distalization. Mbc: mesiobuccal cusp; dbc: distobuccal cusp; mlc: mesiolingual cusp; dlc: distolingual cusp; mra: mesial root apex; dra: distal root apex; cc: center of the crown; rc: center of the root. The data are presented as mean ± standard deviation. A positive value indicates molar extrusion while a negative value indicates molar intrusion. *: Three-dimensional analysis on CBCT.

Time Points	Parameter		Reference	Change (mm)
Post-Distalization	Mandibular First Molar (L6s)	L6mbc	Wu et al. (2021) [31] *	−0.78 ± 0.33
		L6dbc	Wu et al. (2021) [31] *	−0.91 ± 0.31
		L6mlc	Wu et al. (2021) [31] *	−0.56 ± 0.89
		L6dlc	Wu et al. (2021) [31] *	−0.62 ± 0.84
		L6mra	Wu et al. (2021) [31] *	−0.29 ± 1.08
		L6dra	Wu et al. (2021) [31] *	−0.28 ± 0.66
		L6cc	Wu et al. (2021) [31] *	−0.53 ± 1.37
		L6rc	Wu et al. (2021) [31] *	−0.41 ± 0.96
	Mandibular Second Molar (L7s)	L7mbc	Wu et al. (2021) [31] *	−0.81 ± 1.46
		L7dbc	Wu et al. (2021) [31] *	−1.06 ± 0.65
		L7mlc	Wu et al. (2021) [31] *	−0.64 ± 1.19
		L7dlc	Wu et al. (2021) [31] *	−0.72 ± 1.07
		L7mra	Wu et al. (2021) [31] *	−0.30 ± 1.01
		L7dra	Wu et al. (2021) [31] *	−0.27 ± 0.82
		L7cc	Wu et al. (2021) [31] *	−0.59 ± 0.94
		L7rc	Wu et al. (2021) [31] *	−0.56 ± 0.91

**Table 8:** The size of skeletal vertical changes evaluated on the lateral cephalometric radiograph after mandibular molar distalization. SN-MP: sella-nasion-mandibular plane angle, PP-MP: palatal plane-mandibular plane angle, SN-GoGn: sell-nasion-gonion-gnathion angle, FMA (FH-MP): Frankfurt horizontal-mandibular plane angle. The data are presented as mean ± standard deviation or mean [95% confident interval]. A positive value indicates an increase in the mandibular plane angle after treatment, negative value indicates a decrease in the mandibular plane angle after treatment.

Time Points	Parameter	Reference	Change (°)
Post-Distalization	SN-GoGn	Wu et al. (2021) [31] *	1.73 ± 5.37
	SN-MP	Wu et al. (2021) [31] *	−0.99 ± 5.85
	PP-MP	Wu et al. (2021) [31] *	0.66 ± 2.54
	FMA (FH-MP)	Wu et al. (2021) [31] *	1.97 ± 4.58
Post-Treatment	SN-MP	Rota et al. (2022) [6]	0.14 [−3.82, 4.09]
	PP-MP	Rota et al. (2022) [6]	0.81 [−3.95, 5.56]

*: Three-dimensional cephalometric analysis on CBCT.

## Data Availability

The original contributions presented in the study are included in the article, further inquiries can be directed to the corresponding author.
